# Genotype-specific responses in Atlantic salmon (*Salmo salar*) subject to dietary fish oil replacement by vegetable oil: a liver transcriptomic analysis

**DOI:** 10.1186/1471-2164-12-255

**Published:** 2011-05-20

**Authors:** Sofia Morais, Jarunan Pratoomyot, John B Taggart, James E Bron, Derrick R Guy, J Gordon Bell, Douglas R Tocher

**Affiliations:** 1 Institute of Aquaculture, University of Stirling, Stirling FK9 4LA, UK; 2 Landcatch Natural Selection Ltd, The e-Centre, Cooperage Way, Alloa, FK10 3LP, UK

## Abstract

**Background:**

Expansion of aquaculture is seriously limited by reductions in fish oil (FO) supply for aquafeeds. Terrestrial alternatives such as vegetable oils (VO) have been investigated and recently a strategy combining genetic selection with changes in diet formulations has been proposed to meet growing demands for aquaculture products. This study investigates the influence of genotype on transcriptomic responses to sustainable feeds in Atlantic salmon.

**Results:**

A microarray analysis was performed to investigate the liver transcriptome of two family groups selected according to their estimated breeding values (EBVs) for flesh lipid content, 'Lean' or 'Fat', fed diets containing either FO or a VO blend. Diet principally affected metabolism genes, mainly of lipid and carbohydrate, followed by immune response genes. Genotype had a much lower impact on metabolism-related genes and affected mostly signalling pathways. Replacement of dietary FO by VO caused an up-regulation of long-chain polyunsaturated fatty acid biosynthesis, but there was a clear genotype effect as fatty acyl elongase (elovl2) was only up-regulated and desaturases (Δ5 fad and Δ6 fad) showed a higher magnitude of response in Lean fish, which was reflected in liver fatty acid composition. Fatty acid synthase (FAS) was also up-regulated by VO and the effect was independent of genotype. Genetic background of the fish clearly affected regulation of lipid metabolism, as PPARα and PPARβ were down-regulated by the VO diet only in Lean fish, while in Fat salmon SREBP-1 expression was up-regulated by VO. In addition, all three genes had a lower expression in the Lean family group than in the Fat, when fed VO. Differences in muscle adiposity between family groups may have been caused by higher levels of hepatic fatty acid and glycerophospholipid synthesis in the Fat fish, as indicated by the expression of FAS, 1-acyl-sn-glycerol-3-phosphate acyltransferase and lipid phosphate phosphohydrolase 2.

**Conclusions:**

This study has identified metabolic pathways and key regulators that may respond differently to alternative plant-based feeds depending on genotype. Further studies are required but data suggest that it will be possible to identify families better adapted to alternative diet formulations that might be appropriate for future genetic selection programmes.

## Background

Fish are highly nutritious components of the human diet. In addition to providing high quality and easily digested protein, vitamins and minerals, they are particularly important in being the main source of essential n-3 long-chain polyunsaturated fatty acids (LC-PUFA). The beneficial effects of these fatty acids, such as eicosapentaenoic acid (EPA) and docosahexaenoic acid (DHA), include prevention of a range of cardiovascular and inflammatory diseases, and neurological disorders [1]. With catches from commercial fisheries stagnating since 2001, aquaculture is supplying an increasing proportion of fish for human consumption, estimated at around 50% of total supply in 2008 [2]. However, the expansion of aquaculture and the demands it makes upon resources provide many challenges, leading to questions concerning the sustainability of this activity. In particular, marine and salmonid aquaculture relies heavily on fish meal (FM) and fish oil (FO), obtained from wild fishery stocks, for the production of fish feeds and around 88.5% of the total global production of FO is currently used by aquaculture [3]. The increasing scarcity of FO supplies will seriously limit aquaculture growth, and the future of this activity therefore strongly depends on reducing its reliance on FO by seeking to replace them with alternative, largely terrestrial, oils. Vegetable oils (VO) represent a potentially critical resource in this respect. However, VO lack the n-3 LC-PUFA which are abundant in FO, and farming fish on diets containing a high proportion of VO results in lower levels of these omega-3 fatty acids in flesh, compromising their health-promoting effects to the human consumer [4].

The use of selective breeding programs to enhance traits of commercial importance is becoming increasingly more common in aquaculture [5]. Combining genetic selection with changes in commercial feed formulations (i.e., higher levels of inclusion of VO) may be a viable strategy to meet worldwide demand for farmed fish without compromising animal welfare or nutritional value. Recently we showed that deposition and/or retention in flesh of dietary n-3 LC-PUFA, EPA and DHA, is a highly heritable trait in salmon [6], prompting further interest in exploring genotype-nutrient interactions. Other recent work has investigated potential interactions between genetic selection for body fatness and dietary lipid level in rainbow trout [7,8], and the effects of FM and/or FO replacement on the liver transcriptome of both rainbow trout and Atlantic salmon [9-11]. However, there are few data on the interaction between genotype and dietary fatty acid composition. In this respect, microarrays have great potential for application as hypothesis-generating tools. The objective of the present study was to investigate nutrient-genotype interactions in two groups of Atlantic salmon families, Lean and Fat, fed diets where FO was completely replaced by a VO blend. The knowledge gained concerning how this substitution affects hepatic metabolism and, furthermore, how these effects may depend on the genetic background of the fish, not only informs our understanding of lipid metabolism more generally but is also highly relevant to the strategy of genetic selection for families better adapted to alternative and more sustainable feed formulations in the future. A previous study has already focused on hepatic cholesterol and lipoprotein metabolism [12], which was shown to present a significant diet × genotype interaction, while here we will present more broadly the effects of the factors 'diet' and 'genotype'.

## Results

### Microarray results

**Figure 1:**
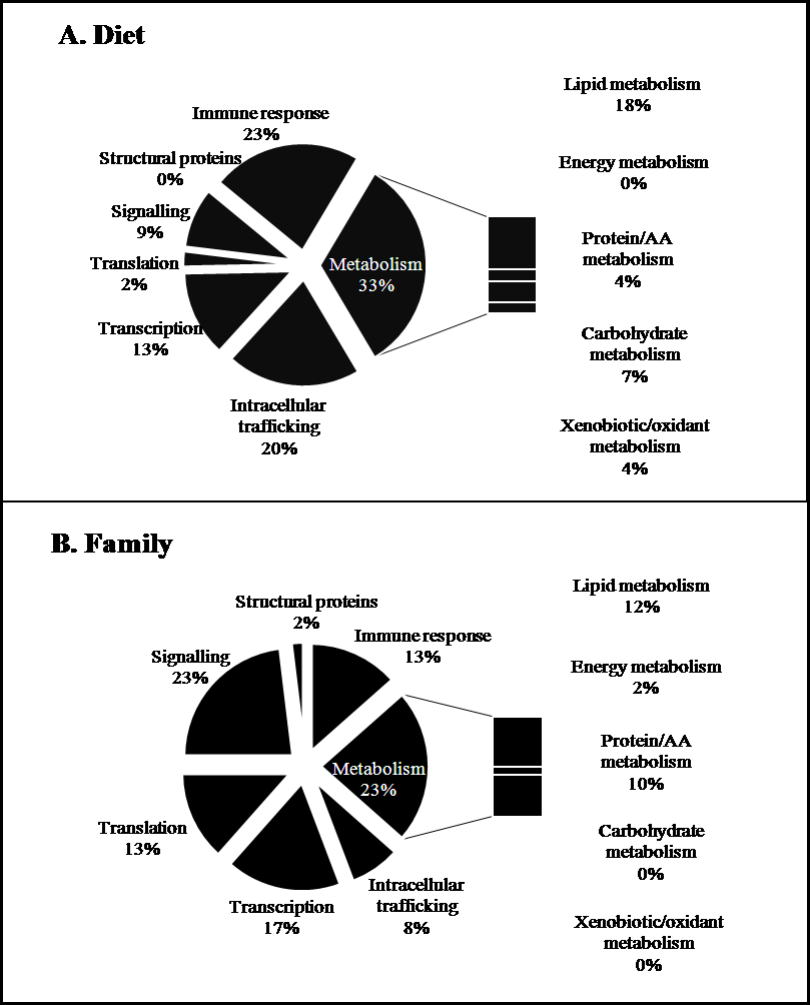
**Functional categories of genes differentially expressed in Atlantic salmon liver**. The top 100 most significant clones (two-way ANOVA analysis; p < 0.05) which were differentially expressed between the two diets (A) and family groups (B) were categorized according to biological function. Non-annotated clones, those representing the same gene or with a miscellaneous function (Tables 2 and 3) are not represented.

Two-way ANOVA of the cDNA microarray dataset returned a high number of features showing evidence of differential expression for each factor - 713 for diet and 788 for genotype - and hence a more detailed analysis was restricted to the top 100 most significant hits for each factor, which were then categorised according to function (excluding 33-35% non-annotated features) (Figure 1). The functional category most affected by diet was that of metabolism (mainly lipid and carbohydrate metabolism), while immune response and intracellular trafficking were also affected. Within lipid metabolism, the affected genes are involved in PUFA, fatty acid and cholesterol biosynthesis (fatty acyl desaturases - Δ5 fad and Δ6 fad, fatty acid synthase - FAS, squalene monooxygenase and possibly cytochrome P450 reductase), glycerophospholipid metabolism (phospholipase D3) and acylglycerol homeostasis (angiopoietin-like 3). Some genes related to carbohydrate metabolism, implicated in glycolysis, glutamine/fructose 6-phosphate and glycerol-3-phosphate metabolism, such as alpha-enolase, glutamine-fructose-6-phosphate transaminase 1(GFPT1) and glycerol kinase, respectively, were also identified as being significantly affected by diet. Genotype had a lower impact on metabolism-related genes (primarily lipid and protein metabolism) and affected mostly genes involved in signalling. Regarding lipid metabolism, primary roles of affected genes are in glycerophospholipid metabolism (N-acylethanolamine-hydrolyzing acid amidase precursor, lipid phosphate phosphohydrolase 2 - LPP2 and 1-acyl-sn-glycerol-3-phosphate acyltransferase - AGPAT), fatty acid transport (intestinal fatty acid binding protein) and lipoprotein metabolism (apolipoprotein B - ApoB and endothelial lipase - EL). In addition, both factors had an effect on a relatively high number of transcription-related genes. Detailed lists of the top 100 most significant genes for diet and genotype, organised by biological function and including the normalised expression ratio between treatments, are shown in Tables 1 and 2, respectively. Gene Ontology enrichment analysis, which enables the identification of GO terms significantly enriched in the input entity list when compared to the whole array dataset, was performed for both factors, providing evidence for which biological processes may be particularly altered in the experimental conditions being compared. For diet, seven significant GO terms, all interrelated, were identified: oxidoreductase activity, stearoyl-CoA 9-desaturase activity, unsaturated fatty acid biosynthetic and metabolic processes, very long chain fatty acid (VLCFA) biosynthetic and metabolic processes. This is explained by the high number of Δ5 fad and Δ6 fad features that were significantly altered when dietary FO was replaced by VO (Table 1). In contrast, no GO terms were significantly enriched in the genotype list.

**Table 1:** Liver transcripts corresponding to the top 100 most significant features exhibiting differential expression between diets

Accession or probe number	Gene	VO/FO		ANOVA p-value
			
		Lean	Fat	
***Metabolism***				
*Lipid metabolism*				
can_D6D_S1B04	Delta-6 fatty acyl desaturase	2.8	1.9	<0.0001
can_D6D_S1B03	Delta-6 fatty acyl desaturase	2.1	1.4	<0.0001
can_D5D_S1B01	Delta-5 fatty acyl desaturase	2.3	1.5	<0.0001
can_D6O_S1B06	Delta-6 fatty acyl desaturase	2.0	1.4	<0.0001
CK887422	Delta-6 fatty acyl desaturase	2.0	1.8	<0.0001
EG647320	Delta-6 fatty acyl desaturase	1.8	1.4	0.0001
CK876943	Fatty acid synthase	1.4	1.6	0.0002
can_D5D_S1B02	Delta-5 fatty acyl desaturase	2.1	1.2	0.0003
CK894344	Phospholipase D3	- 1.1	- 1.2	0.0006
EG647463	Cytochrome P450 reductase	- 1.4	- 1.1	0.0017
liv_lrr_01F07	Angiopoietin-like 3	1.2	1.4	0.0020
can_D5O_S1B05	Delta-6 fatty acyl desaturase	1.5	1.2	0.0045
CK879648	Squalene monooxygenase	1.9	1.1	0.0058
*Protein and amino acid metabolism*				
CK893821	Sequestosome 1or ubiquitin-binding protein P62	- 1.3	- 1.1	0.0022
CK884400	Kynureninase (L-kynurenine hydrolase)	1.3	1.2	0.0065
*Carbohydrate metabolism*				
int_oss_T4F08	Alpha-enolase putative	- 1.2	- 1.0	0.0007
liv_lrr_01A06	Glutamine-fructose-6-phosphate transaminase 1	1.2	1.8	0.0032
CO470771	Glycerol kinase	1.1	1.1	0.0062
*Xenobiotic and oxidant metabolism*				
AJ425332	Thioredoxin domain-containing protein 8	- 1.3	- 1.3	0.0024
EG355339	Glutathione S-transferase A	- 1.4	- 1.4	0.0036
***Transport/intracellular trafficking***				
CK886667	Na/K ATPase	1.5	1.1	<0.0001
CN181143	Coatomer subunit alpha	1.2	1.4	0.0004
DW588711	Synaptic vesicle glycoprotein 2B	1.1	1.1	0.0004
CK894482	Taurine transporter	- 1.3	- 1.3	0.0019
CK887866	ABC-type branched-chain amino acid transport systems ATPase component	1.2	1.1	0.0022
CO470399	Sodium/potassium-transporting	- 1.2	- 1.2	0.0044
EG647422	Transferrin	- 1.2	- 1.1	0.0047
EG648286	ATP-binding cassette sub-family B member 10, mitochondrial	1.2	1.3	0.0055
AM083913	Chromatin modifying protein 2a	- 1.3	- 1.2	0.0068
***Regulation of transcription***				
CK894063	Zinc finger protein 183	- 1.5	- 1.3	0.0003
CK890154	Butyrate response factor 2	1.5	1.1	0.0043
EG648112	Retrovirus-related Pol polyprotein	1.1	1.3	0.0046
CK885196	CCAAT/enhancer binding protein delta1	1.2	1.2	0.0052
CK890573	MADS box protein AP1b	1.2	1.2	0.0053
CK883722	Y-box binding protein	1.1	1.3	0.0067
***Translation***				
AM402452	Phenylalanyl-tRNA synthetase, alpha subunit	1.4	1.9	0.0022
***Signalling/Signal transduction***				
CK886572	GSK-3-binding protein	1.9	1.0	0.0002
CK892148	Growth factor receptor-bound protein 7	- 1.5	- 1.1	0.0028
ova_opk_09K06	Phosphoinositide 3-protein kinase	- 1.1	- 1.3	0.0031
CK873849	Receptor-type tyrosine-protein phosphatase beta precursor	- 1.1	- 1.1	0.0036
DW590775	Myozenin-1	1.6	1.5	0.0055
***Immune response***				
EG647383	Human leukocyte antigen (HLA) class II histocompatibility	1.1	1.2	0.0008
AM402762	Complement component C8 alpha chain	1.3	1.7	0.0018
EG649410	D-dopachrome tautomerase	1.4	1.1	0.0019
AJ425750	Non-histone chromosomal protein H6	1.1	1.3	0.0024
CK884265	Ganglioside GM2 activator	1.3	1.1	0.0030
AM402841	Complement component C8 alpha chain	1.5	2.1	0.0032
CK886548	T cell receptor (TCR)-alpha/delta locus	- 1.2	- 1.0	0.0041
spl_sts_18A08	Leukotriene B4 receptor 1 putative	- 1.4	- 1.1	0.0047
CK880083	Interleukin-15 precursor	1.1	1.3	0.0067
AJ425732	CD97 antigen isoform 2	1.2	1.1	0.0066
***Miscellaneous and unknown function***				
CK897269	Biotinidase precursor	1.3	1.7	0.0002
AM402518	Biotinidase	1.4	1.9	0.0006
CK894173	EFHD2 (EF hand domain containing2)	1.2	1.1	0.0007
kid_cki_A1E04	S100-A1 calcium binding	1.4	1.1	0.0009
CO469646	beta B3-crystallin	- 1.1	- 1.0	0.0012
CO469710	Transmembrane protein 30A	- 1.1	- 1.2	0.0019
AJ425502	Heme oxygenase 1	- 2.7	- 1.7	0.0024
CK885237	EF-hand domain-containing protein D2	1.1	1.1	0.0026
AJ425502	Heme oxygenase 1	- 2.6	- 1.4	0.0037
DW588567	S100 calcium binding protein beta subunit	1.1	1.1	0.0042
CK897725	Type-1 growth hormone	1.1	1.1	0.0053
BM414485	Apoptosis-inducing factor mitochondrion-associated inducer	1.5	1.4	0.0054
BM414504	Syndecan 2	- 1.2	- 1.1	0.0061
BI468143	Anaphase-promoting complex subunit CDC26	- 1.3	- 1.3	0.0064
CK885116	17-beta hydroxysteroid dehydrogenase 13	- 1.1	- 1.4	0.0065

Annotated features (65% of all clones) are arranged by categories of biological function and, within these, by decreasing significance (assessed by two-way ANOVA). Also indicated are the GenBank accession numbers for each clone (or, when not available, the probe number is given instead) and the expression ratio between fish fed VO and those fed FO, for each genotype (Lean and Fat).

**Table 2:** Liver transcripts corresponding to the top 100 most significant features exhibiting differential expression between family groups

Accession or probe number	Gene	Lean/Fat		ANOVA p-value
			
		FO	VO	
***Metabolism***				
*Lipid metabolism*				
CK889835	N-acylethanolamine-hydrolyzing acid amidase precursor	- 1.4	- 1.2	0.0001
can_Apo_S1A12	Apolipoprotein B	- 1.4	- 1.1	0.0014
BM414066	Endothelial lipase precursor	- 1.1	- 1.6	0.0015
CK898924	Lipid phosphate phosphohydrolase 2	- 1.2	- 1.2	0.0017
AJ425826	Intestinal fatty acid binding protein	1.1	1.2	0.0032
CO470953	1-acyl-sn-glycerol-3-phosphate acyltransferase	- 1.1	- 1.3	0.0040
*Energy metabolism/generation of precursor metabolites*				
EG649459	NADH dehydrogenase (ubiquinone) 1 beta subcomplex	1.2	1.2	0.0009
*Protein and amino acid metabolism*				
CK900470	26S protease regulatory subunit 7	- 1.1	- 1.2	0.0020
mus_mfo_15B08	Proteasome subunit alpha type-1	1.2	1.1	0.0021
EG648604	Serine protease-like protein	1.0	1.4	0.0023
CO470297	Transmembrane protease, serine 2	- 1.2	- 1.1	0.0032
DW592216	Ubiquitin carboxyl-terminal hydrolase 5	- 1.1	- 1.1	0.0040
***Transport/intracellular trafficking***				
CK886667	Na/K ATPase	1.1	1.6	<0.0001
CK884193	Polycystin-2 (Polycystic kidney disease 2 protein homolog)	1.1	1.2	0.0002
CK890974	Mitochondrial solute carrier family 25 member 25	- 1.2	- 2.2	0.0002
CK896189	Mitochondrial solute carrier family 25 member 25	- 1.3	- 2.3	0.0002
CK880187	ATP-binding cassette sub-family B member 8, mitochondrial	- 1.3	- 1.1	0.0011
***Regulation of transcription***				
CK881770	Hematopoietically-expressed homeobox protein	- 1.1	- 1.6	0.0001
CK888834	BTEB transcription factor	- 1.4	- 1.5	0.0002
CK895950	Transcription factor CP2-like	- 1.2	- 1.6	0.0010
CK884953	Nuclear transcription factor Y subunit beta	- 1.2	- 1.1	0.0015
CK888548	Rev protein - Human immunodeficiency virus 1	- 1.5	- 1.0	0.0019
CK883410	Retinoic acid receptor gamma (nuclear receptor)	- 1.1	- 1.1	0.0020
int_rpk_78B12	Sp3 transcription factor	- 1.1	- 1.4	0.0035
CK876044	Homeobox protein HoxB13	- 1.2	- 1.2	0.0041
DW589427	Cullin-associated and neddylation-dissociated 1 (CAND1)	1.4	1.1	0.0044
***Translation***				
AJ424434	Ribosome production factor 1	1.1	1.2	0.0002
gil_oss_G6P11	40S ribosomal protein S23	1.3	1.3	0.0008
EG648403	60S ribosomal protein L7a	1.2	1.2	0.0013
CK893177	40S ribosomal protein S26	1.1	1.2	0.0013
DW591137	40S ribosomal protein S3a	- 1.2	- 1.1	0.0024
EG647811	60S ribosomal protein L7	1.1	1.1	0.0026
EG648956	Eukaryotic translation initiation factor 1A	1.1	1.1	0.0033
AJ424851	40S ribosomal protein S18	1.2	1.2	0.0034
***Signalling/Signal transduction***				
CK884714	14-3-3 protein epsilon	1.1	1.1	0.0003
EG648400	Guanine nucleotide binding protein (G protein)	- 1.2	- 1.2	0.0004
CK888542	Insulin-like growth factor binding protein 1	- 1.2	- 2.5	0.0008
CK886572	GSK-3-binding protein putative	- 1.1	1.8	0.0009
CK877143	Calpain-1	- 1.1	- 1.4	0.0010
EG648399	PTC7 protein phosphatase homolog	- 1.2	- 1.1	0.0016
EG648333	Stathmin	1.1	1.2	0.0018
CK898969	G-protein coupled receptor 37	- 1.1	- 1.1	0.0019
DW589782	Amyloid beta (A4) precursor-like protein	1.1	1.2	0.0034
CK898590	Mitogen-activated protein kinase kinase 4	- 1.0	- 1.2	0.0035
CK897997	Calpastatin	- 1.1	- 1.1	0.0039
AJ424385	Protein tyrosine phosphatase, receptor-type, zeta1	- 1.1	- 1.2	0.0041
***Structural proteins***				
AJ425204	Tropomyosin-1 alpha (muscle contraction)	- 1.1	- 1.2	0.0031
***Immune response***				
CK886548	T cell receptor (TCR)-alpha/delta locus	- 1.1	- 1.2	0.0005
CK894741	Complement factor D (adapsin)	- 1.1	- 1.2	0.0007
AM042439	Major histocompatibility complex (MHC) class I antigene	- 1.3	- 1.4	0.0014
kid_cki_A1G02	Interferon-inducible protein	- 1.2	- 1.4	0.0015
CO471904	Major histocompatability complex (MHCI)	- 1.1	- 1.2	0.0019
CK894557	Major histocompatability complex (MHCI)	- 1.2	- 1.1	0.0023
AM042249	Major histocompatability complex (MHCI)	- 1.1	- 1.2	0.0025
bra_opk_01B08	Scavenger receptor cysteine-rich gene	- 1.3	- 1.6	0.0029
CO469739	T-cell receptor(TCR)-alpha/delta locus	- 1.1	- 1.1	0.0030
AJ424124	Major histocompatibility complex (MHC class I)	- 1.2	- 1.1	0.0037
hrt_opk_07E23	Interferon alpha 1-like	- 1.1	- 1.1	0.0041
***Miscellaneous and unknown function***				
CK898014	Protein fuzzy homolog	- 1.1	- 1.2	0.0002
kid_cki_A2A05	Cyclin B2	- 1.3	- 1.4	0.0006
EG647643	Purine nucleoside phosphorylase	- 1.1	- 2.0	0.0010
liv_lrr_07B04	Nuclear protein 1	- 1.1	- 2.1	0.0028
AM402622	Non-POU domain containing, octamer-binding	- 1.7	- 1.4	0.0029
EG648147	Adhesion-regulating molecule 1	- 1.2	- 1.1	0.0037
BM414485	Apoptosis-inducing factor mitochondrion-associated inducer	1.4	1.5	0.0038
DW589496	Cyclin-dependent kinase inhibitor	- 1.2	- 1.3	0.0044

Annotated features (67% of all clones) are arranged by categories of biological function and, within these, by decreasing significance (assessed by two-way ANOVA). Also indicated are the GenBank accession numbers for each clone (or, when not available, the probe number is given instead) and the expression ratio between Lean and Fat fish fed either FO or VO.

### RT-qPCR

Quantification of gene expression by RT-qPCR was performed to partially validate the microarray results and to examine particular genes of interest in detail. The latter included several fatty acyl desaturase and elongase genes involved in the LC-PUFA biosynthesis pathway that were identified by GO analysis as being significantly affected by diet, as well as peroxisome proliferator-activated receptors (PPAR) and sterol regulatory element binding protein 1 (SREBP-1), which have important roles in regulating the expression of multiple lipid metabolism genes (Table 3). In spite of the generally low fold changes, a good correspondence in terms of expression ratios or in the direction of change (up- or down-regulation), was obtained between the microarray and RT-qPCR results for most quantified genes, including Δ5 fad and Δ6 fad, FAS and heme oxygenase 1 (HOX) for the factor diet, and ApoB, LPP2 and AGPAT for the factor genotype (Tables 1, 2, 3). However, comparison of the microarray and RT-qPCR expression results show an inverse change in expression for GFPT1 and glutathione S-transferase A (GST) in response to diet, the latter only in the Fat group, and of EL between family groups, although only when feeding on FO (where the fold-change in the microarray was negligible). Nonetheless, a perfect match was not expected given that RT-qPCR primers were obtained either from published work (e.g., GST) or, when available, designed on well characterized sequences such as GenBank reference sequences or clusters on the gene index database for Atlantic salmon (ASGI), which do not necessarily match exactly the clone on the array. In fact, in the case of EL there is evidence that the microarray probe has high similarity with multiple EST's and hence is likely to have resulted in cross-hybridisation [12], while the reference sequence for GFPT1 and the clone in the microarray are only 93% identical in the aligned region.

**Table 3:** Relative expression of genes assayed by RT-qPCR in liver of Atlantic salmon

	VO/FO				Lean/Fat			
		
	Lean		Fat		FO		VO	
				
Genes	Ratio	p-value	Ratio	p-value	Ratio	p-value	Ratio	p-value
Δ5 fad	**3.95**	**0.001**	**2.04**	**0.002**	**-2.33**	**0.009**	-1.20	0.317
Δ6 fad_a	**8.27**	**0.000**	**4.52**	**0.004**	**-1.85**	**0.049**	-1.02	0.942
elovl5a	1.18	0.505	-1.03	0.817	-1.18	0.420	1.03	0.908
elovl5b	1.57	0.184	1.05	0.758	-1.25	0.471	1.19	0.416
elovl2	**2.35**	**0.025**	-1.04	0.841	-1.56	0.098	1.58	0.112
FAS	**1.76**	**0.005**	**2.11**	**0.003**	**-1.72**	**0.001**	**-2.04**	**0.011**
PPARα	**-2.22**	**0.000**	1.10	0.643	-1.16	0.358	**-2.86**	**0.001**
PPARβ	-1.92	0.161	1.56	0.169	1.24	0.659	**-2.44**	**0.002**
PPARγ	-1.10	0.828	-1.67	0.251	-2.00	0.214	-1.32	0.229
SREBP-1	-1.16	0.761	**3.32**	**0.004**	1.82	0.332	**-2.13**	**0.022**
GST	-1.18	0.412	**1.40**	**0.010**	1.29	0.210	**-1.28**	**0.028**
HOX	-2.69	0.132	**-1.82**	**0.013**	1.83	0.271	1.24	0.120
GFPT1	-1.33	0.244	-1.65	0.090	-1.18	0.619	1.05	0.783
ApoB	1.40	0.443	1.84	0.152	-1.15	0.791	-1.52	0.076
EL	**3.52**	**0.034**	**8.57**	**0.002**	1.38	0.494	-1.75	0.115
LPP2	-1.33	0.506	-1.31	0.606	-1.22	0.754	-1.25	0.516
AGPAT	1.40	0.375	**1.42**	**0.041**	-1.05	0.906	-1.07	0.574

Values are normalised (by *cofilin-2*) gene expression ratios between fish fed VO in relation to FO for each family group or of Lean fish in relation to the Fat group when fed either one of the diets. Values in bold are significantly different, at p < 0.05 (REST 2008).

fad: fatty acyl desaturase (Δ5 and Δ6); elovl: elongase (three different transcripts); FAS: Fatty acid synthase; PPARs; peroxisome proliferator-activated receptors (three isoforms); SREBP-1: Sterol regulatory element binding protein-1; GST: glutathione S-transferase A; HOX: heme oxygenase 1; GFPT1: glutamine-fructose-6-phosphate transaminase 1; ApoB: apolipoprotein B; EL: endothelial lipase; LPP2: lipid phosphate phosphohydrolase 2; AGPAT: 1-acyl-sn-glycerol-3-phosphate acyltransferase.

In terms of regulation of gene expression by the factor diet, the qPCR results confirmed the significant up-regulation of Δ5 fad and Δ6 fad in fish fed VO, with a higher fold change being measured for Δ6 fad. In addition, the expression ratio was higher in the Lean family group than in Fat fish, as had also been indicated in the microarray analysis. Of the elongase genes, only elovl2 was significantly up-regulated by the VO diet, but just in the Lean family group. Furthermore, quantification of PPAR genes revealed that only PPARα was down-regulated significantly when salmon were fed the VO diet, but only in the Lean family group. On the other hand, expression of SREBP-1 was only significantly affected in Fat fish, being up-regulated in fish fed the VO diet. Other genes which were significantly and consistently regulated were FAS and EL (both up-regulated when VO replaced FO in the diet), while GST, HOX and AGPAT only showed significant regulation in Fat fish. Finally, comparison between the two family groups showed a significantly lower expression of Δ5 fad, Δ6 fad, PPARα, PPARβ, SREBP-1 and GST in the Lean group but only when fish were fed FO, in the case of fads, or when fed the VO diet, in the case of PPARs, SREBP-1 and GST. In addition, FAS was also significantly down-regulated in the Lean group, independent of diet.

### Liver fatty acid composition

Fatty acid analysis of liver showed significant differences in all fatty acid classes related mostly to diet but also to genotype (except for total n-3 PUFA and total PUFA) (Table 4). The percentage of total n-6 PUFA (reflecting mainly 18:2n-6) was significantly increased when VO replaced FO in the diet. Levels of total n-3 PUFA were, on the other hand, significantly higher in the FO treatments independent of genotype. For EPA and DHA there was a significant diet × genotype interaction, resulting from the fact that, when comparing Fat and Lean fish, higher levels of these LC-PUFA were found in the Fat family group when fed the FO diet but the inverse was observed when the same fish were fed the VO diet.

**Table 4:** Liver fatty acid composition (percentage of total fatty acids) of Atlantic salmon Lean and Fat family groups fed diets containing either FO or VO

Parameters	FO		VO		ANOVA		
		
	Fat	Lean	Fat	Lean	Diet	Genotype	Diet×Gen
Fatty acid							
Total saturated	25.8 ± 0.8	22.1 ± 1.5	19.1 ± 1.0	18.3 ± 1.0	<0.0001	<0.0001	0.0044
Total monoenes	23.8 ± 1.3	31.1 ± 2.5	40.8 ± 3.3	39.0 ± 2.5	<0.0001	0.0141	0.0002
18:2n-6	2.9 ± 0.1	3.0 ± 0.3	10.1 ± 0.2	10.4 ± 0.2	<0.0001	0.0317	ns
18:3n-6	0.1 ± 0.1	0.1 ± 0.1	0.1 ± 0.1	0.1 ± 0.0	ns	ns	ns
20:2n-6	0.5 ± 0.0	0.7 ± 0.0	1.6 ± 0.2	1.9 ± 0.1	<0.0001	<0.0001	ns
20:3n-6	0.4 ± 0.0	0.4 ± 0.0	1.3 ± 0.1	1.4 ± 0.1	<0.0001	ns	ns
20:4n-6	2.2 ± 0.2	1.9 ± 0.2	1.2 ± 0.1	1.4 ± 0.1	<0.0001	ns	0.0009
22:5n-6	0.2 ± 0.0	0.2 ± 0.1	0.1 ± 0.0	0.1 ± 0.0	<0.0001	ns	ns
Total n-6 PUFA	6.3 ± 0.3	6.3 ± 0.4	14.4 ± 0.3	15.3 ± 0.3	<0.0001	0.0031	0.0031
18:3n-3	1.0 ± 0.0	1.1 ± 0.2	4.3 ± 0.2	4.7 ± 0.2	<0.0001	0.0021	0.0466
18:4n-3	0.4 ± 0.1	0.4 ± 0.1	0.3 ± 0.0	0.3 ± 0.1	0.0104	ns	ns
20:3n-3	0.2 ± 0.0	0.3 ± 0.0	0.7 ± 0.1	0.7 ± 0.3	<0.0001	ns	ns
20:4n-3	1.6 ± 0.1	2.3 ± 0.3	1.1 ± 0.1	1.3 ± 0.1	<0.0001	<0.0001	0.0021
20:5n-3	8.6 ± 0.3	8.2 ± 0.4	4.7 ± 0.4	5.2 ± 0.4	<0.0001	ns	0.0085
22:5n-3	3.6 ± 0.2	4.4 ± 0.3	1.9 ± 0.2	2.1 ± 0.1	<0.0001	<0.0001	0.0025
22:6n-3	28.6 ± 1.0	23.7 ± 1.9	12.6 ± 1.5	13.1 ± 1.1	<0.0001	0.0011	0.0002
Total n-3 PUFA	44.1 ± 1.1	40.5 ± 1.5	25.7 ± 2.4	27.4 ± 1.4	<0.0001	ns	0.0009
Total PUFA	50.4 ± 1.2	46.8 ± 1.5	40.2 ± 2.6	42.8 ± 1.6	<0.0001	ns	0.0004

Results are means ± SD (n = 6) and p-values of two-way ANOVA are presented for factors 'diet', 'genotype' and interaction between both factors. ns, not significantly different (p > 0.05).

## Discussion

In the present study we analysed the effects of diets containing high levels of plant proteins and with complete replacement of FO by VO on the liver transcriptome of Atlantic salmon, which is the primary metabolic organ of fish, as well as the influence of genotype on these responses. Here we focus on the separate effects of diet and genotype given that interactions, indicating pathways that were differentially affected by diet depending on the genetic background of the fish, were discussed in detail previously [12].

A common methodological difficulty in this type of nutritional experiment is that effects are typically quite subtle although physiological and metabolic pathways can be impacted by even small fold changes in gene expression. This has been demonstrated by several studies [7,9,11] and by previously reported data from the present study showing that low fold changes in gene expression were associated with biochemical differences in tissue lipid class and apolipoprotein composition [12]. Furthermore, low fold changes observed in this study were generally corroborated by RT-qPCR, even if the low expression ratios meant that differences were not always significant. It should also be noted that a total match between the microarray and the RT-qPCR results is not expected due to the approach taken to design RT-qPCR primers on better annotated reference sequences rather than on less well characterized microarray clones. In view of the whole genome duplication event that occurred in salmonid fishes [13], transcriptomic and gene expression studies are often more challenging due to the presence of duplicated and highly similar genes whose transcripts might be differentially regulated, as observed previously for lipoprotein lipase [12]. Therefore, collectively, and in conjunction with previous studies, data obtained in the present microarray study enabled identification of pathways that may be differentially affected by both dietary oil composition and genetic background related to flesh adiposity.

### Effects of diet on lipid metabolism

Within the list of genes affected by diet, those involved in fatty acyl desaturation were prominent, leading to the identification, through GO enrichment analysis, of several terms related to LC-PUFA biosynthetic and metabolic processes. The up-regulation of Δ5 fad and Δ6 fad in both family groups when dietary FO was replaced by VO was confirmed by RT-qPCR. Several studies have previously demonstrated up-regulation of genes involved in LC-PUFA biosynthesis in salmon when FO is replaced by VO [10,14,15]. RT-qPCR also confirmed previous work showing that elovl2 is responsive to dietary n-3 LC-PUFA levels [15], being the only elongase whose expression was up-regulated when FO was replaced by VO. However, a significant effect was only observed in the Lean family group. In addition, both microarray and RT-qPCR analyses indicated that the up-regulation of Δ5 fad and Δ6 fad showed a considerably higher fold-change in the Lean fish, due mainly to lower basal expression of fads in Lean salmon, compared to Fat, when fed FO. These results indicate that the activity of this biosynthetic pathway may be dependent on the genetics of the fish, with different family groups showing differences in the magnitude of response. The liver fatty acid composition revealed that differences in EPA and DHA levels between fish fed either diet were smaller in the Lean fish, due to higher n-3 LC-PUFA in fish fed VO and lower n-3 LC-PUFA in fish fed FO, compared to the equivalent treatments in the Fat group. In addition, intermediates in the biosynthetic pathway, such as 20:4n-3 and 22:5n-3, tended to be present at higher levels in the Lean family group, suggesting that differences observed in the levels of mRNA of LC-PUFA biosynthesis genes, which have been shown to correlate with the enzymatic activity of this pathway in salmon [16,17], were reflected in biochemical composition.

Another lipid metabolism gene significantly affected by diet was FAS, which was up-regulated in both family groups when fed VO. A well demonstrated effect of dietary FO supplementation in mammals is hypotriglyceridemia, resulting from a coordinated effect of n-3 LC-PUFA in suppressing hepatic lipogenesis and enhancing fatty acid oxidation in liver and muscle [18]. Furthermore, this gene also appears to be regulated at a pre-translational level and hence changes in FAS transcription are likely to result in important effects in terms of enzyme activity [19]. Similar mechanisms are believed to operate in fish but, although reduced hepatic lipogenic activity modulated by LC-PUFA has been demonstrated *in vitro *[20], a direct relationship with dietary FO and VO has not always been clear *in vivo *[21,22]. The regulation of FAS in response to FO replacement by VO did not show an interaction with the flesh leanness/fatness phenotype in this study, as might have been expected. This was because genotype also had a significant effect, with the Lean group having lower levels of FAS expression than the Fat fish, with a similar fold-change in both diets.

Regulation of lipid metabolism is complex and controlled by several transcription factors and nuclear receptors, including PPARs and SREBPs. SREBP-1c is a major regulator of lipogenesis in mammals [18]. Here we measured the expression of SREBP-1 as there is no evidence for the existence of alternatively spliced isoforms in salmon, and primers corresponded to an identical region in mammalian SREBP-1a and SREBP-1c [23]. Our results agree with Minghetti et al. [23], who showed SREBP-1 was increased by cholesterol and decreased by EPA and DHA supplementation in a salmon cell line, denoting a similar nutritional regulation to mammals [18]. However, there was a clear genetic effect as expression of SREBP-1 was 3-fold higher in Fat salmon fed VO, containing lower EPA, DHA and cholesterol, than in fish fed FO, whereas no regulation was observed in the Lean group.

PPARs have been less studied in fish than in mammals but present evidence suggests PPARα and PPARβ have similar ligands and functions to their mammalian homologues, while PPARγ may present some functional differences [24,25]. LC-PUFA are well recognised enhancers of PPARα activity in fish, and while the response of PPARβ to LC-PUFA might be variable between fish species, an enhancement of activity in sea bass, plaice and sea bream [24-26] and of expression in Atlantic salmon [27] has been observed. In addition, and unlike rodents, PPARα and PPARβ have a similar pattern of expression in response to fasting and feeding in sea bream liver, indicating that they may be regulated similarly [25]. In the present study, PPARα was down-regulated when VO replaced FO but only in the Lean family group and, although not statistically significant, PPARβ showed a similar trend, suggesting similar transcriptional regulation of these nuclear receptors by dietary fatty acid composition. These results thus indicate that the genetic background of the fish might affect PPAR transcriptional responses to LC-PUFA. In contrast, no nutritional regulation was observed for PPARγ transcription in liver, in accordance with previous studies in fish, including salmon, and its predominant role in adipocytes [24,28].

The hypotriglyceridemic effects of n-3 LC-PUFA in mammals involve activation of PPARα, leading to up-regulation of β-oxidation genes (including carnitine palmitoyltransferase I - CPT1 and acyl-CoA oxidase - ACO) and suppression of SREBP-1c transcription that down-regulates lipogenic enzymes [29,30]. As previously reported, FAS expression was up-regulated in both family groups fed the VO diet but neither CPT1 nor ACO expression, was affected [12]. As elovl2 expression was only altered in the Lean fish and both Δ5 fad and Δ6 fad showed greater up-regulation in Lean salmon fed VO, we may speculate that PPARα (and potentially also PPARβ) expression may be involved in down-regulation of LC-PUFA biosynthesis. Paradoxically, fatty acyl desaturases are regulated by both SREBPs and PPARs in mammals [31]. In addition, PPARα agonists regulate the transcriptional activity of elongases in rat, although only elovl5 and not elovl2 [32]. However, in mammals, PPARα ligands induce the transcription of elongases and desaturases while we observed an up-regulation of elovl2 and a stronger stimulation of Δ5 fad and Δ6 fad transcription when PPARα expression was lower. In the rat and human Δ6 fad gene promoters, both PUFA and PPARα response regions have been identified which suppress and induce, respectively, Δ6 fad expression [33]. The molecular mechanisms of transcriptional regulation of these genes are complex and will require further investigation in salmon [34]. In contrast, target genes of SREBP-1 remain elusive and, although it may regulate FAS expression [23], this was only observed in Fat fish whereas, in the Lean group, another mechanism is required to explain up-regulation of FAS in VO-fed fish as expression of SREBP-1 was unaffected. Nonetheless, the action of SREBP-1 is under the regulation of liver X receptor (LXR) and these complex pathways have only recently started to be investigated in fish [23].

Another gene affected by diet was squalene epoxidase (SQLE), which was up-regulated by VO but only markedly in the Lean family group. This enzyme catalyses the first oxygenation step in sterol biosynthesis, a pathway identified earlier as presenting a diet **× **genotype interaction [12]. In contrast, cytochrome P450 reductase (CPR) was down-regulated in salmon fed VO, particularly in Lean fish. This enzyme has multiple roles as the electron donor for several oxygenase enzymes, such as cytochrome P450 (involved in drug and xenobiotic metabolism, and sterol and bile acid synthesis), HOX and cytochrome b5 (which supports both sterol and LC-PUFA biosynthesis pathways). In addition, it has key roles in the biosynthesis of several signalling factors and the regulation of oxidative response genes [reviewed by [35]]. CPR is transcriptionally regulated by PPARα in mouse and, given the comparable PPARα and CPR expression in Lean salmon fed VO, similar regulation likely occurs in salmon. However, changes in CPR expression can be related to several processes that were affected by FO replacement. Thus, CPR expression could be linked to changes in both cholesterol and LC-PUFA biosynthesis, both more marked in Lean fish, although this is unlikely because VO induced up-regulation of these pathways. A more likely association is with cell oxidant metabolism, also suggested by the microarray results as being possibly down-regulated in VO-fed fish. In particular, down-regulation of HOX in salmon fed VO, more marked for Lean fish correlating with CPR expression, might be an indication of this.

### Effect of diet on carbohydrate and intermediate metabolism

Within the metabolism genes that were identified by the microarray analysis as being significantly affected by dietary oil substitution, a few relate to carbohydrate metabolism, particularly glucose and intermediary metabolism. Given that similar effects were observed in previous salmonid studies, and that a few signal transduction genes present in the list of diet significant effects are also potentially implicated in these pathways, these results warrant further discussion, even if the observed fold changes were low. An association between lipid and carbohydrate metabolism in salmon is not surprising given that the pathways of lipogenesis, lipolysis, glycolysis, gluconeogenesis and pentose phosphate shunt are all interrelated in the regulation of body energy homeostasis. In mammals, the role of LC-PUFA as "fuel partitioners" involves both directing fatty acids away from anabolic and towards catabolic routes as well as enhancing glucose flux to glycogen, mediated by effects on SREBP-1 and transcription factors that regulate key genes of lipid metabolism and glycolysis [30]. Similar mechanisms may operate in fish but differences are likely given that carnivorous fish like salmon have low capacity to use carbohydrate and appear to show features of glucose intolerance [36,37]. Nonetheless, dietary n-3/n-6 ratio has been shown to influence mRNA levels of the glucose transporter GLUT4 in Atlantic salmon muscle, with some reflection in plasma glucose [38]. In addition to a decreased hexokinase and phosphoenolpyruvate carboxykinase expression, complete replacement of FM and FO by vegetable alternatives in rainbow trout resulted in a slightly increased expression of glycerol kinase, as observed here [11]. This enzyme is at the intersection of lipid-carbohydrate metabolism and over-expression of this gene in human muscle and rat hepatoma cells resulted in higher TAG synthesis and up-regulation of the pentose phosphate pathway providing reducing power for lipogenesis [39]. Panserat et al. [11] hypothesised that the up-regulation of glycerol kinase may be related to higher lipid biosynthesis in liver when trout were fed plant-based diets. Similarly, our results, associated with the observed changes in FAS mRNA when VO replaced FO, suggest a possible relationship with lipogenesis. Also possibly related with this was the up-regulation of two different biotinidase clones with the potential to increase availability of substrates for FAS and/or gluconeogenesis in VO-fed fish. This gene, besides being involved in the regulation of gene expression, including genes of glucose metabolism, codes for an enzyme that recycles biotin, which is a co-factor for several carboxylases responsible for production of substrates for lipogenesis and gluconeogenesis [40].

Another gene affected by diet was alpha-enolase, which was slightly down regulated in Lean fish fed VO. A similar effect was observed in liver of salmon fed rapeseed oil in comparison to FO [9]. This glycolytic enzyme participates in the conversion of glucose to pyruvate, a key intermediate at the intersection of multiple metabolic pathways, including lipogenesis. Thus, this might result in lower levels of pyruvate for conversion to acetyl-CoA in VO-fed fish. This result does not necessarily conflict with an increase in lipogenesis given that, in fish, carbon skeletons for *de novo *fatty acid production are mainly derived from amino acid catabolism rather than from carbohydrates, whose main contribution towards lipogenesis is to supply NADPH via the pentose-phosphate pathway [37].

Finally, a few signalling genes that were significantly affected by diet might also have an effect on glucose metabolism, assuming that similar cascades exist in fish. One of these is phosphoinositide 3-protein kinase (PI3K), which mediates insulin's effects on glucose, lipid and protein metabolism, and that was significantly down regulated in VO-fed fish. Among other roles, it regulates glucose cellular uptake in mammals, recruiting GLUT4 transporters to the cell surface [41]. In addition, it is found upstream of a signal transduction cascade regulating glycogen synthesis through glycogen synthase, by inactivating glycogen synthase kinase-3 (GSK3) [41,42]. In our study, expression of GSK3-binding protein (GBP) was significantly increased in VO-fed Lean fish. GBP is a protein that blocks GSK3, which in turn inactivates glycogen synthase [43]. Hence, it is possible that the oil composition of the diet might also affect glucose metabolism and glycogen storage.

### Effect of diet on oxidative stress and immune response

Increased oxidative stress associated with the consumption of FO has been typically reported in fish and mammals [27,44,45]. Accordingly, genes related to oxidant metabolism were found in the significant list for diet. A thioredoxin domain-containing protein, possessing an antioxidant role [46], and GST, which detoxifies peroxidised lipids and xenobiotics [47], were down-regulated in salmon fed VO, consistent with the higher auto-oxidative potential of LC-PUFA in FO. However, quantification of GST by RT-qPCR was not consistent with the microarray result, although the possibility exists that different GST genes with differential regulation exist in salmon and this requires clarification. In addition, the observed down-regulation of HOX in VO-fed fish, validated by RT-qPCR, might be related to a decrease in oxidative stress in these fish. This enzyme catalyses the degradation of heme and can be induced by oxidative stress [48] and may be increased during pro-inflammatory states, being thought to increase resistance to oxidative injury and ameliorate inflammation [49]. The n-3 LC-PUFA in FO have important anti-inflammatory actions in mammals [50], which does not correlate with the expression of HOX and its putative role in inflammation in this case. Inflammation is an important mechanism in immune defence but, in fish, the demonstrated effects of LC-PUFA on immune and inflammatory mechanisms have been inconsistent [45]. However, a recent study has clearly shown an effect of dietary oil composition on the progression of a myxosporean parasite infection in Gilthead sea bream, with fish fed the VO diet showing higher signs of the disease and faster course of infection in comparison with those on a FO diet [51]. On the other hand, the synthesis of pro-inflammatory eicosanoids was increased in the intestine of salmon fed vegetable-based diets in response to acute stress [52]. In the present study immune response was the second highest category of genes affected by diet, after metabolism. Whether this is due to the potential anti-inflammatory role of dietary FO or whether VO diets can have detrimental health effects is not clear as the fold-changes were subtle, as expected in unchallenged animals. Nonetheless, the majority of genes related to processes of both innate and adaptive immunity were up-regulated in fish fed VO. Only T-cell and leukotriene B_4 _(LTB_4_) receptors, that are reduced after antigen and LTB_4 _exposure, respectively, and, in the case of LTB_4 _receptor, increased after EPA administration [53-55], were down-regulated in salmon fed VO.

### Differences in gene expression between Lean and Fat genotypes

Muscle adiposity is a trait of great importance in animal production, aquaculture included, and hence physiological changes induced by genetic selection for this phenotype have been examined in various animals, including rainbow trout [7,8]. In the present study the main differences between family groups were associated with signal transduction pathways, followed by metabolism. Only a small number of lipid metabolism genes varied in relation to muscle adiposity, as reported previously in rainbow trout, where the main differences were related to lipogenesis and mitochondrial oxidative metabolism [7,8]. In our study glycerophospholipid metabolism may have been down-regulated in the Lean family group through AGPAT and LPP2, two enzymes acting consecutively on *de novo *TAG and phospholipid biosynthesis [56,57]. Quantification of AGPAT and LPP2 expression by RT-qPCR confirmed this down-regulation but fold-changes were too subtle to be significant. AGPAT converts lysophosphatidic acid into phosphatidic acid (PA), while LPP2 then catalyzes the conversion of PA to diacylglycerol. All these molecules can function as second messengers and are involved in the regulation of multiple signalling pathways. Therefore, down-regulation of this pathway in the Lean group has the potential to lower lipid biosynthesis, at least partly explaining the flesh lipid phenotype, but may also alter levels of lipid signalling molecules. On the other hand, differences in muscle adiposity might also be caused by higher hepatic "de novo" fatty acid synthesis in the Fat family group, as indicated by the expression of FAS. In a previous study, no differences were found in the expression of ACO and CPT1, which suggested that the phenotypes could not be explained by differences in β-oxidation [12]. By contrast, in rainbow trout Fat and Lean families, β-oxidation and mitochondrial oxidative metabolism, but not lipogenesis, were affected by genetic selection [7], although another study using the same trout lines suggested differences related to lipogenesis rather than fatty acid oxidation [8]. Thus, both metabolic processes are likely involved and discrepancies in the data are likely due to lack of methodological sensitivity to detect the small fold-changes that are possibly characteristic of these biological processes and typical in this type of experiment.

PPARα, PPARβ and SREBP-1 were also regulated in response to genotype, being down-regulated in Lean fish, but only when fed the VO diet. In cobia, *Rachycentron canadum*, a negative correlation was found between PPARα mRNA levels in liver and body lipid deposition [58]. Furthermore, PPARβ appears to play a similar role in fish to that in mammals, as a ubiquitous regulator of fat burning and with a role in energy mobilisation during early development [24,25]. Therefore, both PPARα and PPARβ might have a role in the control of adipogenesis in fish and it may be the case that, similarly to chickens [59], Fat salmon might have higher lipid turnover than their Lean counterparts when fed a diet that predisposes for hepatic fat deposition, even though the end result is higher lipid accumulation in liver [60]. To explain this, Collin et al. [59] suggested that a fat chicken family is better "equipped" to deal with higher circulating levels of TAG when fed a high fat diet, compared to lean chicken. On the other hand, we observed a direct relationship between SREBP-1 and FAS expression in the Fat family group in response to diet, as well as in VO-fed fish in response to genotype. It thus appears that SREBP-1 may be partly responsible for higher lipogenesis in Fat fish, compared to Lean, when fed VO.

## Conclusions

This study has enabled the identification of metabolic pathways and key regulators that may respond differently to more sustainable diets, in which FO is replaced by VO, depending on genotype, thus confirming the potential of microarrays as hypothesis-generating tools, even in these nutritional studies where changes in gene expression are quite subtle. Collectively, and in conjunction with previous studies, the data indicate that dietary lipid composition may potentially affect glucose, glycogen storage and intermediary metabolism, in addition to lipogenesis, supporting a role for LC-PUFA in "fuel partitioning" in fish as well as in mammals. Therefore, more integrative studies investigating the effects of dietary VO on energy homeostasis are required. However, important genotype-related differences may also exist in the regulation of metabolism. In terms of lipid metabolism, expression of LC-PUFA and lipid biosynthesis genes, as well as of key regulator transcriptional factors, was differentially affected by diet depending on the genetic background of the fish. Although further studies are required, the present data indicate that it will be possible to identify families better adapted to alternative diet formulations that might be appropriate for future genetic selection programmes.

## Methods

### Feeding trial and sampling

A dietary trial was conducted using two genetically characterised and contrasting groups of farmed Atlantic salmon post-smolts, comprising full-sib families selected from the Landcatch Natural Selection Ltd (LNS) breeding program (Argyll, Scotland). The choice of the two family groups was based on estimated breeding values (EBVs) of the parents for high or low flesh adiposity, assessed by Torry Fatmeter (Distell Industries, West Lothian, UK), a trait that was found to have a heritability ranging from 0.17 to 0.39 in this dataset. The two groups were created from four unrelated full-sib families; two families from the extreme lower end of the EBV distribution for flesh lipid content ('Lean') and two families from the extreme upper end of the distribution ('Fat'). The average EBV for the lipid content of the two Fat families was 2.00 percentage units higher than that of the two selected Lean families, representing a standardised selection differential of 2.33 standard deviations. Assessment of the flesh and viscera lipid content at the end of the feeding trial confirmed differences in adiposity between the two genotypes, in spite of an interaction with diet being also found [12].

Two thousand fish of each group were stocked into eight 12 × 5 m^3 ^net pens at the Ardnish Fish Trials Unit (Marine Harvest Scotland, Lochailort, Highland; 500 fish pen^-1^). Duplicate pens from each group of fish were fed one of two experimental diets (Skretting ARC, Stavanger, Norway) containing 32-25% fish meal, 40-45% plant meals and 27.5-30% oil supplied either as northern fish oil (FO) or as a vegetable oil (VO) blend comprising rapeseed, palm and *Camelina *oils in a ratio of 5:3:2 [12]. Diets were formulated to fully satisfy the nutritional requirements of salmonid fish [61] and contained similar levels of PUFA (around 31%) but different n-3 and n-6 PUFA contents, 25.3% and 4.6% in the FO diet and 13.4% and 17.1% in the VO diet, respectively. Further details including full diet formulations, proximate and fatty acid compositions of the feeds can be found in Bell et al. [60].

After 55 weeks on the experimental diets 25 fish were sampled per pen. The fish were killed by a blow to the head following anaesthesia using MS222, 24 h after the last meal. Samples of liver were immediately frozen on dry ice and stored at -70°C for molecular and fatty acid analyses.

### RNA extraction and purification

Liver tissue (0.2 g) from six individuals per experimental group was rapidly homogenised in 2 mL of TRI Reagent (Ambion, Applied Biosystems, Warrington, U.K.) using an Ultra-Turrax tissue disrupter (Fisher Scientific, Loughborough, U.K.) and stored at -70°C. Total RNA was later isolated, following manufacturer's instructions, and RNA quality (integrity and purity) and quantity was assessed by gel electrophoresis and spectrophotometry (NanoDrop ND-1000, Thermo Scientific, Wilmington, U.S.A.). One hundred micrograms of total RNA from each individual sample was further cleaned by mini spin-column purification (RNeasy Mini Kit, Qiagen, Crawley, UK), and then re-quantified and quality assessed as above.

### Microarray hybridizations and image analysis

The TRAITS/SGP (v.2.1) salmon 17 k cDNA microarray, described in detail by Taggart et al. [10], was used in this experiment (ArrayExpress accession: A-MEXP-1930). A dual-label experimental design was employed for the microarray hybridisations. Each experimental sample was competitively hybridised against a common pooled-reference sample, which comprised equal amounts of all samples used in the study. This design permits valid statistical comparisons across all treatments to be made. The entire experiment comprised 24 hybridisations - 2 genotypes (Lean/Fat) × 2 diets (FO/VO) × 6 biological replicates.

An indirect labelling methodology was employed in preparing the microarray targets. Antisense amplified RNA (aRNA) was produced from 500 ng of purified total RNA per sample using the Amino Allyl MessageAmpTM II aRNA Amplification Kit (Ambion, Applied Biosystems), as per manufacturer's instructions, followed by Cy3 or Cy5 fluor incorporation mediated by a dye-coupling reaction, as previously described in detail [12]. Experimental samples and the pooled reference sample (batch reaction) were labelled with Cy3 and Cy5 dye suspension stocks (PA23001 or PA25001, GE HealthCare, Little Chalfont, UK), respectively. Unincorporated dye was removed by column purification (Illustra AutoSeq G-50 spin columns; GE Healthcare). Dye incorporation and aRNA yield were quantified by spectrophotometry (NanoDrop ND-1000) and further quality controlled by separating 0.4 μL of the sample through a thin mini-agarose gel and visualising products on a fluorescence scanner (Typhoon Trio, GE Healthcare).

Microarray hybridisations were performed in a Lucidea semi-automated system (GE Healthcare), without a pre-hybridisation step. For hybridisation of each array, each labelled biological replicate and corresponding pooled reference (40 pmol each dye, c. 150 ng aRNA) were combined and added to the hybridisation solution, comprising 185 μL 0.7X UltraHyb buffer (Ambion), 20 μL poly(A) at l0 mg/mL (Sigma-Aldrich, Dorset, UK), 10 μL herring sperm at c. 10 mg/mL (Sigma-Aldrich) and 10 μL ultra pure BSA at 10 mg/mL (Sigma-Aldrich), as detailed previously [12]. Two post-hybridisation automatic washes followed by six manual washes to a final stringency of 0.1× SSC (EasyDipTM Slide staining system; Canemco Inc., Quebec, Canada) were performed before scanning.

Scanning was performed at 10 μm resolution using an Axon GenePix 4200AL Scanner (MDS Analytical Technologies, Wokingham, Berkshire, U.K.) with laser power constant (80%) and "auto PMT" enabled to adjust PMT for each channel such that less than 0.1% of features were saturated and that the mean intensity ratio of the Cy3 and Cy5 signals was close to one. BlueFuse software (BlueGnome, Cambridge, U.K.) was then used to identify features and extract fluorescence intensity values from the resultant TIF images. Following a manual spot removal procedure and fusion of duplicate spot data (BlueFuse proprietary algorithm), the resulting fluorescence intensity data and quality annotations for the 17,102 gene features, were exported into the GeneSpring GX version 10.0.2 analysis platform (Agilent Technologies, Wokingham, Berkshire, U.K.) after undergoing a block Lowess normalisation. Data transformation and quality filtering were then performed and all control features were excluded from subsequent analyses [12]. This returned a list of 14,772 genes eligible for statistical analysis. Experimental annotation complied fully with minimum information about a microarray experiment (MIAME) guidelines [62]. The experimental hybridisations and further methodological details are archived on the EBI ArrayExpress database (http://www.ebi.ac.uk/arrayexpress/) under accession number E-TABM-1089.

### RT-qPCR

Expression of selected genes was determined by reverse transcription quantitative real time PCR (RT-qPCR). Details on the target qPCR primer sequences are given in Table 5. In addition, amplification of three potential reference genes - *cofilin-2*, elongation factor-1α (*elf-1α*) and *β-actin *- was performed. However, only *cofilin-2 *expression proved to be sufficiently stable across treatments for normalisation of the results. *Cofilin-2 *had been established in a previous salmon cDNA microarray study as a suitable reference gene on the basis of constant expression between FO and VO based feeds over a wide range of time points ('unidentified liver EST', [10]).

**Table 5:** Primers used for RT-qPCR analyses

Transcript	Primer sequence (5'-3')	Fragment	Ta	Efficiency	Accession No.	Source
Δ5fad	GTGAATGGGGATCCATAGCA	192 bp	56°C	0.995	AF478472 ^1^	[66]
	AAACGAACGGACAACCAGA					
Δ6fad_a	CCCCAGACGTTTGTGTCAG	181 bp	56°C	0.944	AY458652 ^1^	[66]
	CCTGGATTGTTGCTTTGGAT					
elovl5a	ACAAGACAGGAATCTCTTTCAGATTAA	137 bp	60°C	0.925	AY170327 ^1^	[15]
	TCTGGGGTTACTGTGCTATAGTGTAC					
elovl5b	ACAAAAAGCCATGTTTATCTGAAAGA	141 bp	60°C	0.940	DW546112 ^1^	[15]
	CACAGCCCCAGAGACCCACTT					
elovl2	CGGGTACAAAATGTGCTGGT	145 bp	60°C	0.960	TC91192 ^2^	[15]
	TCTGTTTGCCGATAGCCATT					
FAS	GTGCCCACTGAATACCATCC	212 bp	60°C	0.995	CK876943 ^1^	New design
	ATGAACCATTAGGCGGACAG					
PPARα	TCCTGGTGGCCTACGGATC	111 bp	60°C	0.986	DQ294237 ^1^	[67]
	CGTTGAATTTCATGGCGAACT					
PPARβ	GAGACGGTCAGGGAGCTCAC	151 bp	60°C	0.992	AJ416953 ^1^	[67]
	CCAGCAACCCGTCCTTGTT					
PPARγ	CATTGTCAGCCTGTCCAGAC	144 bp	60°C	0.999	AJ416951 ^1^	[67]
	TTGCAGCCCTCACAGACATG					
SREBP-1	GCCATGCGCAGGTTGTTTCTTCA	151 bp	63°C	0.942	TC148424 ^2^	[20]
	TCTGGCCAGGACGCATCTCACACT					
GST	ATTTTGGGACGGGCTGACA	81 bp	60°C	0.989	GE619558 ^1^	[68]
	CCTGGTGCTCTGCTCCAGTT					
HOX	GTCAACGCATCACCCTTCTT	206 bp	60°C	0.997	BT046987 ^1^	New design
	ATGGGGTCCTTCATCCTCTT					
GFPT1	GTGGTTTGGCAGACCTCCTA	177 bp	60°C	0.999	NM_001140266 ^1^	New design
	TGTACGGTGCCATCTTTCAA					
ApoB	AGCCTTCGATGCTGTCGGCCA	153 bp	60°C	1.000	TC79364 ^2^	[12]
	AGGAGCACAGGCAGGGTGGTT					
EL	CCGGTGCTGCTGGAGGAAGC	378 bp	60°C	0.962	NM_001140535 ^1^	[12]
	CGACATGCAGGTCATCGGT					
LPP2	TCCGGAAGAACTCGCAATAC	174 bp	60°C	0.949	NM_001140716 ^1^	New design
	ACATCACGTCCACCAAGACA					
AGPAT	GAGAGCCAGAGGTTGAGGTG	245 bp	60°C	0.941	NM_001141753 ^1^	New design
	CAGAGTGAAGGCGATGTGAA					
						
Reference genes:						
elf-1α	CTGCCCCTCCAGGACGTTTACAA	175 bp	60°C	0.986	AF321836 ^1^	[15]
	CACCGGGCATAGCCGATTCC					
β-actin	ACATCAAGGAGAAGCTGTGC	141 bp	56°C	0.968	AF012125 ^1^	[15]
	GACAACGGAACCTCTCGTTA					
Cofilin-2	AGCCTATGACCAACCCACTG	224 bp	60°C	0.999	TC63899 ^2^	[15]
	TGTTCACAGCTCGTTTACCG					

^1 ^GenBank (http://www.ncbi.nlm.nih.gov/)

^2 ^Atlantic salmon Gene Index (http://compbio.dfci.harvard.edu/tgi/)

For RT-qPCR, 1 μg of column-purified total RNA per sample was reverse transcribed into cDNA using the VersoTM cDNA kit (ABgene, Surrey, U.K.), following manufacturer's instructions, using a mixture of random hexamers (400 ng/μL) and anchored oligo-dT (500 ng/μL) at 3:1 (v/v). Negative controls (containing no enzyme) were performed to check for genomic DNA contamination. A similar amount of cDNA was pooled from all samples and the remaining cDNA was then diluted 20-fold with water. RT-qPCR analysis used relative quantification with the amplification efficiency of the primer pairs being assessed by serial dilutions of the cDNA pool. qPCR amplifications were carried out in duplicate (Quantica, Techne, Cambridge, U.K.) in a final volume of 20 μL containing either 5 μL or 2 μL (for the reference genes and HOX) diluted (1/20) cDNA, 0.5 μM of each primer and 10 μL AbsoluteTM QPCR SYBR^® ^Green mix (ABgene). Amplifications were carried out with a systematic negative control (NTC-non template control). The qPCR profiles contained an initial activation step at 95°C for 15 min, followed by 30 to 40 cycles (depending on target): 15 s at 95°C, 15 s at the specific primer pair annealing temperature (Ta; Table 5) and 15 s at 72°C. After the amplification phase, a melt curve of 0.5°C increments from 75°C to 90°C was performed, enabling confirmation of the amplification of a single product in each reaction. RT-qPCR product sizes were checked by agarose gel electrophoresis and the identity of amplicons of newly designed primers (FAS, GFPT1, HOX, LPP2 and AGPAT) was confirmed by sequencing.

### Lipid extraction and fatty acid analyses

Total lipids from six fish per treatment were extracted and determined gravimetrically from 1-2 g of liver by Ultra Turrax homogenisation in 20 volumes of chloroform/methanol (2:1 v/v) [63]. Fatty acid methyl esters (FAME) were prepared by acid-catalysed transesterification of total lipids [64]. Following purification, FAME were separated and quantified by gas-liquid chromatography using a Thermo Fisher Trace GC 2000 (Thermo Fisher, Hemel Hempstead, UK) equipped with a fused silica capillary column (ZB wax, 30 m×0.32 mmi.d.; Phenomenex, Macclesfield, UK) with hydrogen as carrier gas and using on-column injection. The temperature gradient was from 50 to 150°C at 40°C/min and then to 195°C at 1.5°C/min and finally to 220°C at 2°C/min. Individual methyl esters were identified by comparison with known standards. Data were collected and processed using the Chromcard for Windows (version 2.00) computer package (Thermoquest Italia S.p.A., Milan, Italy).

### Statistical analysis

Microarray hybridisation data were analysed in GeneSpring GX version 10.0.2 (Agilent Technologies) by two-way ANOVA, which examined the explanatory power of the variables 'diet' and 'genotype' (diet×genotype interaction presented in [12]), followed by Gene Ontology (GO) enrichment analysis, at a significance level of 0.05. No multiple test correction was employed as previous analyses, confirmed by RT-qPCR, indicate that such corrections are over-conservative for this type of data [14]. Gene expression results assessed by RT-qPCR were analysed by the ΔΔCt method using the relative expression software tool (REST 2008, http://www.gene-quantification.info/), employing a pair wise fixed reallocation randomisation test (10,000 randomisations) with efficiency correction [65], to determine the statistical significance of expression ratios between two treatments. Finally, significant differences in liver fatty acid composition were determined by means of two-way ANOVA, at a significance level of p < 0.05, using the Graphpad Prism™ (version 4.0) statistical package (Graphpad Software, San Diego, CA).

## Competing interests

The authors declare that they have no competing interests.

## Authors' contributions

SM and JP performed laboratory analyses and data analysis; DRG was responsible for family selection; JBT and JEB supported the microarray analysis; SM wrote the first draft of the manuscript, followed by contributions from remaining authors; SM, JGB and DRT planned and coordinated the research; DRG, JGB and DRT were project leaders. All authors read and approved the final manuscript.
